# Toward Trusted IoT by General Proof-of-Work

**DOI:** 10.3390/s23010015

**Published:** 2022-12-20

**Authors:** Chih-Wen Hsueh, Chi-Ting Chin

**Affiliations:** 1 Department of Computer Science and Information Engineering, National Taiwan University, Taipei 10617, Taiwan; 2 Department of Risk Management and Insurance, Ming Chuan University, Taipei 11103, Taiwan

**Keywords:** IoT, blockchain, finality, consensus, PoW, PoPT, PowerTimestamp, synchronization, energy, metaverse

## Abstract

Internet of Things (IoT) is used to describe devices with sensors that connect and exchange data with other devices or systems on the Internet or other communication networks. Actually, the data not only represent the concrete things connected but also describe the abstract matters related. Therefore, it is expected to support trust on IoT since blockchain was invented so that trusted IoT could be possible or, recently, even metaverse could be imaginable. However, IoT systems are usually composed of a lot of device nodes with limited computing power. The built-in unsolved performance and energy-consumption problems in blockchain become more critical in IoT. The other problems such as finality, privacy, or scalability introduce even more complexity so that trusted IoT is still far from realization, let alone the metaverse. With general Proof of Work (GPoW), the energy consumption of Bitcoin can be reduced to less than 1 billionth and proof of PowerTimestamp (PoPT) can be constructed so that a global even ordering can be reached to conduct synchronization on distributed systems in real-time. Therefore, trusted IoT is possible. We reintroduce GPoW with more mathematic proofs so that PoPT can be optimal and describe how PoPT can be realized with simulation results, mining examples and synchronization scenario toward trusted IoT.

## 1. Introduction

Since the invention of the vending machine in the 1980s, the Internet of Things (IoT) has been a concept to bring computers, networks, and machines to improve our daily life. Many hardware, software and networking technologies such as Radio Frequency IDentification (RFID), Near Field Communication (NFC), Wireless Sensor Network, Cloud computing, Edge computing, etc. [1,2] are emerged, forming various IoT platforms, and successfully reach different goals, such as the smart home. Basically, IoT is used to describe devices (mostly embedded systems) with sensors that connect and exchange data with other devices or systems on the Internet or other communication networks. Actually, the data not only represent the concrete things such as temperature or time at the devices connected but also describes the abstract matters related such as security or privacy. Therefore, it is expected to support trust on IoT since blockchain was invented so that trusted IoT could be possible. As a trust machine, blockchain needs to support trust in all aspects such as security, privacy, transparency, efficiency, justice, etc. As Augmented Reality, Virtual Reality, and Mixed Reality become more mature, people even imagine and develop platforms and applications for the metaverse recently. Beyond IoT, which integrates computers with real world, metaverse goes to the virtual universe created in computers. Trusted IoT is the key component to connect the real world and virtual “universe” to realize all the applications. Otherwise, metaverse is just an augmented game.

**Figure 1:**
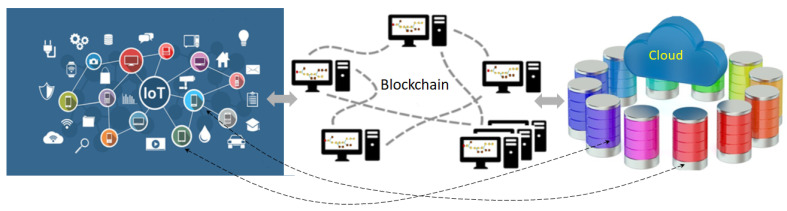
Network architecture for blockchain-based IoT.

However, IoT systems are usually composed of a lot of device nodes with limited computing power. The built-in and unsolved performance and energy-consumption problems in blockchain become more critical in IoT. The other problems such as finality, privacy, or scalability introduce even more complexity, so that trusted IoT is still far from realization. Even blockchain technologies are not ready for trusted IoT; many researchers work from different perspectives to support the trust, in part. As shown in Figure 1, G. Wang et al. [3] proposed a hierarchical storage architecture to store recent blocks from the IoT network in the blockchain network and the rest in cloud to tackle the scalable storage problem in blockchain. This can be a good example of the IoT blockchain network. As the nodes in Internet can be autonomous, each node can be part of the different networks to support more than one services. Actually, IoT nodes can communicate with the cloud nodes directly. It incurs more synchronization and security problems. B. Yu, etc. [4] has studied how and why to build IoT blockchain. It is based on Hyperleger Fabric [5], a permissioned blockchain approach, where nodes need permission to participate the operation of blockchain. Usually, permissioned blockchains are more efficient and a certain percentage of the nodes needs to be trustful. However, it is not secure if there are too many malicious nodes. Gong-Guo and Zhou [6] develop a dynamic security authentication management on chain codes (smart contract [7] of Hyperleger fabric) for IoT environment. However, it is not scalable because permissioned blockchains usually need more message communication to reach consensus. Since IoT is to bring computers, networks, and machines to improve our daily life, for trusted IoT, public blockchain is necessary at least for the inclusion of permissionless users, and it also needs to support trust, security, privacy, flexibility and scalability for various user requirements such as low cost and high efficiency. Supporting only trust in part is far from enough. No previous work can provide a total solution. The fundamental problems are trustful efficient consensus and distributed synchronization in blockchain.

To reach consensus in distributed computing is for the coordinating processes to agree on some data value needed during computation to achieve overall system reliability in the presence of a number of faulty or malicious processes. There are a lot of studies and applications trying to solve different problems of reaching consensus. Main consensuses and more definitions of basic terms used in blockchain can be found in an analysis paper [8]. Efforts on public blockchain can only achieve probabilistic (or stochastic) finality with huge energy consumption or unfair capitalism, while efforts on permissioned application can achieve absolute finality; but, they are private blockchains and are not trustful enough. In a fully asynchronous message-passing distributed system, in which at least one process may have a crash failure, it has been proven in FLP impossibility [9], with initials of authors Fischer, Lynch, and Paterson, resulting that a deterministic algorithm for achieving consensus is impossible. The FLP theorem states that in an asynchronous network where messages may be delayed but not lost, there is no consensus algorithm that is guaranteed to terminate in every execution for all starting conditions, if at least one node may experience failure. However, we still would like to make it possible under some reasonable conditions. While real world communications in systems are often inherently asynchronous, it is more practical and often easier to model as synchronous systems, given that an asynchronous system naturally involves more issues to solve than a synchronous one [10]. The consensus mechanism plays the most important role in blockchain to support trust. However, with the requirements of decentralization, it is not a trivial problem in public blockchain, where permissionless and asynchronous are in nature because anyone can participate at any time without permission.

To reach consensus, it includes the first phase to collect data from peer nodes and the second phase to interpret the data as some proof for confirmation, supporting consensus independently in each node. In the public blockchain, data are collected from permissionless nodes. For being trustful, the data might be generated with much effort and it might need many times of confirmation. In private blockchain, data are collected from permissioned nodes with enough trustful ones. The confirmation can be conducted efficiently. We assume consortium blockchain is a private blockchain with big enough trustful nodes; regardless, they might be out of trust. It is in nature that consensus cannot be reached at once in a distributed system, resulting in partitions sometimes. Each partition stands for the nodes with a different state of the system. This is the trilemma in the distributed system, where consistency, availability and partition tolerance cannot be met at the same time, and in the so-called CAP, with initials of consistency, availability, and partition tolerance, theory [11]. Therefore, for a blockchain to be scalable with more users, nodes, or transactions, according to the CAP theory, there might be more chances of problems occurred with availability, consistency and partitioning. This is also why scalability is difficult in blockchain.

There are also efforts to invent new consensus and a blockchain design for IoT to solve the scalability problem. IOTA [12], just a name but not an acronym, is the first new blockchain design specialized for IoT. IOTA’s main component is the Tangle, a guided acyclic graph (DAG) for transaction storage. A transaction needs to be verified by two randomly chosen previous transactions, and there is no transaction fee. It is claimed scalable and can be fully decentralized to reach a million transactions per second (TPS) if a centralized coordinator for the balancing verification process is removed after popular adoption. Shabandri and Maheshwari [13] use IOTA to enhance IoT security and privacy and discuss a smart utility meter system and a smart car transaction system connected to the internet through a Low Power Wide Area IoT networks. Since IOTA is not popular yet and the coordinator is not removed, it is questionable how can IOTA be scalable in such systems with high demand of TPS. There might be other design problems so that IOTA cannot be popular, e.g., the zero-transaction fee design might result in overloading. Let that alone solve security and privacy.

Other approaches modifying existing technologies continue. The Mystiko [14] blockchain proposed a new federated consensus implemented using the Cassandra Paxos consensus [15] and Apache Kafka [16], which were scalable for big data. However, the paxos consensus does not consider malicious peer nodes. The Tikiri platform [17] is built based on Hyperledger Fabric and Mystiko blockchains for resource-constrained IoT devices. It is evaluated with a Raspberry-Pi based test-bed but the same problems above still remain. Among many IEEE standards published for blockchain recently, IEEE Std 2144.1™ identifies the common building blocks of the framework that blockchain enabled during IoT data lifecycle including data acquisition, processing, storage, analyzing, usage/exchange and obsoletion, and the interactions among these building blocks [18]. Although the standard is not yet the detailed design definition, it is important to reduce the design complexity of IoT applications on the blockchain. Actually, the blockchain standards themselves are still under construction because some fundamental problems are still not solved in distributed systems.

Although none of the efforts above solve all the problems, they own enough users or market share to survive with different advantages, regardless the notorious energy consumption in public blockchain or possible collusion in private blockchain. However, if we cannot trust some IoT nodes or it is permissionless for the inclusion in public, the permissioned approaches above are still not satisfactory. Actually, most of the problems come from the stochastic finality from previous solutions that cannot provide distributed synchronization efficiently and effectively. Common solutions are waiting longer or using multiple resources, ignoring possible attacks or problems in real use. It usually results in various exceptions and even more transactions or synchronization load to handle. Using the epoch, several synchronized block intervals and deterministic finality can be achieved with more flexibility than absolute finality [19]. However, it needs a long (100-block) epoch and uses a distributed hash table to assume the perfect (under some bound) network connection [20]. In reality, the O(1) complexity is difficult to achieve in the jittering network environment and it might not be flexible and trustful enough to impose business logic or reflect user investment.

Suppose the deterministic finality and synchronization problems above are solved in a real-time manner. The scalability problem might also be solved no matter how complex the system is by proper load partitioning in parallel (sharding) and merging hierarchically. Since the resources for solving scalability are limited, any scalability is also limited. For example, network bandwidth might not be increased on demand in real-time accordingly. Therefore, the number of transactions need to be controlled. If most of the transactions are independent or less critical, we can bundle or process them in batch to release the problem. Many transactions or data together in the IoT network can be represented as a hash to send to the blockchain network for trust processing. The complex computing for business logic, privacy or security servers, or huge storage can be left to IoT network or cloud services, as in Figure 1. Actually, the IoT network can represent any existing systems. That is to say, by separating the IoT network and blockchain network with proper synchronization, the limited resource of IoT devices is no longer a constraint for trusted IoT. New applications can be implemented in IoT network and linked to the blockchain network by hashes or be implemented in the blockchain network as a service using smart contracts. IoT network consists of both thin embedded systems and powerful servers with huge data to be processed in real-time. Hot blockchain data can be keep in the blockchain network and cold data can be stored to the cloud as the low-storage design [21] of Ko and etc. A proper or intelligent transaction fee design economically and flexibly might be a necessary component to the success of trusted IoT. If we can find a consensus mechanism with flexible deterministic finality so that the distributed synchronization is possible for blockchain transactions with various business logic, the problems above might be solvable mostly step by step. We can also simplify the complexity of understanding and developing blockchains in this perspective instead of following various technologies with just temporary solutions.

OurChain [22] based on Bitcoin [23] PoW (Proof-of-Work) was prototyped as a public blockchain with a consensus called Proof-of-PowerTimestamp (PoPT) based on EPoW (Estimable PoW) [24]. PowerTimestamp compares the timestamp first then estimated the computing power. More introduction of blockchain chaining, PoW, EPoW, PowerTimestamp, finality, and OurChain can be found in Section 2. Extending from EPoW, GPoW (General PoW) is a general PoW, constructing the trust model of the blockchain with closed-form formula to estimate the computing power to any given level of coefficient of variation. GPoW supports a distributed synchronization solution and solves the energy consumption problem of PoW. Using GPoW, the energy consumption of Bitcoin can be reduced to less than 1 billionth and PoPT becomes feasible. The trilemma in a distributed system also becomes solvable by synchronization in a real-time manner. When enough PoW is collected, GPoW can be conservative to broadcast a block right away or aggressively collecting more data for higher estimated computing power. With time as higher priority, using the conservative GPoW might be preferable for IoT systems with limited computing power. The closed-form formula can help to adjust the system behavior, dynamically adapting network jitter in real-time for reaching the distributed synchronization. By GPoW, a flexible deterministic finality can be achieved by dynamically adjusting the target value of the mining difficulty for the different user behavior. The user behavior can be also modeled by functions of estimated computing power economically and flexibly, as used in EPoW, solving the Sybil attack.

We have introduced aggressive GPoW in the paper “Toward Blockchain Realization” [25]. Without the mathematic model of conservative GPoW, GPoW could only be executed by approximation heuristics using aggressive GPoW. With the mathematic model of conservative GPoW in this paper, optimal design of PowerTimestamp and PoPT are feasible. In addition, distributed synchronization, deterministic finality and even global event ordering can be implemented in blockchain flexibly for general applications. To the best of our knowledge, this is a breakthrough. Actually, the conservative GPoW and Aggressive GPoW are equivalent statistically. We might extend to apply GPoW for trusted Internet in general instead of just IoT. However, in the broader context of the Internet, nodes might have enough computing power so that nodes would like to spend more time for higher rewards. When earlier time is not the higher priority, it might be advantageous for aggressive GPoW if the target value is small. Although in the long run, they are statistically equivalent, manipulation in the short run might not be solvable technically when the target value or reward can be also manipulated easier in the general Internet. Distributed synchronization and global event ordering might be able to slow down manipulation because we believe evil cannot prevail. However, this is not like IoT with the fact of limited computing power, so that earlier time and less computing power is always the higher priority; we can use the formula to provide controllable bounded errors in the solutions of the problems above. Trusted Internet in general might need to wait until trusted IoT is realized and prevails cross chain so that the malicious priority scheme cannot survive. Therefore, we focus on the trusted IoT in this paper and provide more complete introduction, correction, and proof for GPoW, PowerTimestamp and PoPT. With simulation for conservative GPoW on OurChain, the TPS can reach 3000. Our contribution in this paper can be summarized, as follows:

1. We modify a simple and effective network architecture to be used in trusted IoT for any existing systems or new applications on blockchain.
2. We first propose a simple definition for absolute, deterministic, stochastic, and probabilistic finalities. It can help to understand the concept of finality for blockchain.
3. We reintroduce GPoW with errors corrected and first present the mathematic model of conservative GPoW. Actually, conservative GPoW and aggressive GPoW are equivalent statistically, supporting the various needs for trusted IoT.
4. With conservative GPoW, we first find that the optimal design of PowerTimestamp and PoPT are feasible deterministically with a consistent priority scheme, where the earlier time is with the higher priority.
5. We first, in detail, present the PowerTimestamp and the PoPT consensus mechanism with mining examples. Using GPoW, the PowerTimestamp and PoPT support deterministic finality and global event ordering so that distributed synchronization is feasible for general applications. This is a breakthrough.
6. We first introduce a common synchronization scenario in finance, which is difficult to be solved in public blockchain without distributed synchronization.

Section 2 introduces GPoW-related issues. GPoW is introduced in Section 3 with simulation results and comparisons to other main consensuses. PoPT, mining examples and a synchronization scenario are introduced in Section 4. The paper is concluded in Section 5.

## 2. Background

Since blockchain is an emerging technology, we discuss different descriptions and myths on the features of blockchain in the previous paper [25]. We elaborate on GPoW-related issues on IoT in this Section.

### 2.1. Blockchain Chaining

In blockchain, each block is usually identified by the hash of its header. The header contains the ID of previous block, the hash of all transactions, and other information of the block. The ID of the previous block in each block forms a hash chain. The hash chain provides the immutability of blockchain but not trust because incorrect information hashed is still not trustful. The previous block confirmed is called the parent block and the parent of the previous one is called the grandparent block. The value of the cryptographic hash used in blockchain is commonly assumed to be unique and random. Bitcoin wins trust because the blocks mined are “randomly” conducted by the hash-based PoW in a decentralized manner. The more computing power, the easier to mine a block fairly, on average, for any node. If more than one block is mined, broadcasted, and then received in different nodes, including self-mined, only the first verified block (still random chosen) is confirmed, with the ancestor blocks and other information corrected if not consistent. Then, it is appended as a parent block for mining the next one, possibly creating or merging partitions.

Bitcoin mining with the same parent forms a partition. The longest chain or main chain is the chain of blocks in each partition built with the most number of hashes or the largest difficulty accumulated since genesis. The difficulty of each partition is adjusted every 2016 blocks to keep a block mined in an average of 10 min, linear to the total computing power in the partition. As long as the result is random by any permissionless node to mine a block, the difficulty does not have to be high. It is so high because nodes compete to have higher computing power than others. Different partitions are unlikely to have the same difficulty after adjustment and blocks might not be mined and confirmed in each partition. Therefore, most of the time, there will only be one main chain. A Bitcoin block is mature if it has 100 blocks confirmed after itself. Only the mature blocks in the main chain can spend their reward. In this way of decentralization and partition tolerance, no other consensus mechanism ever provides the randomness, as well as trust, better than Bitcoin. Even when there were Bitcoin-related attacks, the Bitcoin core itself has only one split and was restored soon in 2013 due to an undiscovered inconsistency between two versions [26]. For distributed systems as blockchain, partitioning is inevitable as long as system states are changing, no matter which consensus mechanism is used. How the partitions are merged results in different kinds of finality and trust.

### 2.2. Proof-of-Work (PoW)

PoW is a form of cryptographic proof, in which one party (the prover) proves to others (the verifiers) that a certain amount of a specific computational effort has been expended for reaching some consensus, the first phase. Verifiers can subsequently confirm this expenditure with minimal effort on their part [27], in the second phase. PoW is represented by a piece of data sent from a requester to the PoW service provider. It was first proposed to prevent junk mails [28] by doing some significant computing work before sending the email. Bitcoin was not the first try based on PoW [29], but it is the first successful hash-based PoW used in blockchain to generate data for the collection to reach consensus. The PoW is to prove that enough computing work has been conducted so that the winner can append a new block in the blockchain. Some reward could be given to the winner after the block is really confirmed. Since a block takes chances to be confirmed and it might not be really confirmed due to the stochastic finality, the process is called mining, and those mining nodes or people in charge are called miners. Mining with reward as bitcoins in Bitcoin is an incentive for miners to maintain the operation of blockchain, but not necessary. The energy consumption problem using PoW does not come from enough work required for PoW but from the competition of getting the reward as much as possible. The reward becomes very attractive because of the jumping prices of bitcoins, due to the limited supply of bitcoins and too centralized holding. The same principle requiring a certain amount of “work” applies to other consensuses such as proof of space, proof of bandwidth, and proof of ownership as well. However, the proof of stake is different in that it just needs to provide a more deterministic proof of “wealth”, e.g., “age” of coin or how long a coin has been created [24], so that the consensus can be reached efficiently. However, it might be difficult to keep the same level of randomness, permissionlessness, or trust as a PoW.

### 2.3. Estimable Proof-of-Work (EPoW)

Based on PoW, EPoW, the US patented [30] records the highest and lowest hash values to estimate how many trials of nonces are in each mining as an indicator of the estimated computing power of the individual mining nodes. By EPoW, the computing power of the mining nodes can be estimated so that we can reject the blocks mined from the nodes with too small or too big computer power. Therefore, the nodes with big computing power can be discouraged to reduce energy consumption or speculation can also be reduced for the nodes with limited computing power. Although this might increase the number of nodes with computing power in a predefined range or Sybil attack, it can be mitigated economically by decreasing the mining reward or imposing a mining fee for each node.

### 2.4. PowerTimestamp

Time is a fair metric to define the order of events. Since there is no global clock in distributed systems, it is impossible to be sure of the time on a different machine at a different location. Even though we have corrected the time of the clock in each machine, the clocks run in slight different rates, resulting in time drift. The time drift might be minutes a day for different personal computers without further correction periodically. Moreover, it also takes time to obtain the time or correct the time. That is to say, there is no absolute global event ordering in distributed systems. Only partial ordering or casual ordering is possible, where Lamport has explained in 1978 [31] and proposed a solution called the logical clock or Lamport Timestamp [32] later. Since the time scale of the logical clock might be twisted to form the partial order, it is not natural to what we feel on the wall clock and it is not popularly implemented. Without global event ordering, distributed systems still can refer to a centralized clock or adopt partial ordering to conduct the synchronization as long as the conflicts or attacks are acceptable. Actually, most users overlook affordable risks until it is out of control. To be really trusted and scalable by machines without human interference or accumulating drift errors such as IoT, deterministic global event ordering, with controllable bounded errors, is necessary.

Time looks like a concept in our mind to tell the change of the outside world. We record certain moments of time as a timestamp or a period of time by the difference of timestamps. Clocks or watches are just instruments for us to feel time is running, record time easier, and know what time it is. We know timing would be changed by speed from the theory of relativity but what is time or how time is running exactly still remains a secret. The timestamps recorded by different people or at different places might not represent the same concept of time. However, the error of timestamps in a distributed system can be estimated robustly in a given confidence interval [33], even though there are outliers from malicious attackers. With Network Time Protocol (NTP) [34], it can usually maintain the time error to within tens of milliseconds (ms) over the public Internet, and can achieve better than one-ms accuracy in local area networks under ideal conditions. In addition, asymmetric routes and network congestion can cause errors of 100 ms or more.

Therefore, we can estimate the error using the NTP above, called the TimeError, in a distributed system, accurately enough for blockchain down to one-tenth second or less, assuming that dedicated network and flow control are used for the applications not tolerating the network congestion, such as bank operations. For other cases, we can assume that the TimeError of the local area network has the same accuracy. With TimeError, when an event is issued, the timestamp of the event and the estimated computing power is combined as a unique metric, called PowerTimestamp. A global event ordering can be formed such that the event wins if its timestamp is TimeError earlier; otherwise, the event with the higher priority on the indicator wins, assuming both events have the same TimeError. That is, if we cannot tell exactly which event is earlier, we decide by a unique indicator. We assume the higher estimated computing power has the higher priority to boost economy. Note that the higher estimated computing power might have the lower priority for some kind of society fairness. The PowerTimestamp was first implemented in OurChain such that the TimeError is half of the block interval, 1 s roughly for convenience, and the indicator is the estimated computing power by EPoW. The priority of the indicator might be converted so that it is not linear to the estimated computing power or even inverted for any considerations such as energy consumption, speculation, or business model for a certain kind of fairness. For example, if the lower estimated computing power has the higher priority, it might be fairer with more inclusion since the higher computing power already has advantages with the earlier timestamp.

However, for a different area, networks, or groups, the time error might be different. It is not necessary to define a global TimeError for all. There can be different TimeErrors for different needs similar to use different NTP servers. There is no problem to use different TimeErrors in different applications, respectively. Can we still fairly define two PowerTimestamps with different TimeErrors in order when they meet? Yes. At first, the two TimeErrors need to have the same priority scheme, e.g., the smaller value has the higher priority. For example, if the time difference is larger than the big TimeError, the earlier wins. If it is smaller than the small TimeError or in between, the indicators still can tell with certain comparison functions as long as they are defined deterministically with the fairness and keeping the same global event ordering, especially following the transitive law. If there are conflicts, global event ordering should have a higher priority than fairness; otherwise, there might be deadlocks. TimeError is dependent on network delay but not on estimated computing power. With higher computing power, the timestamp tends to be earlier. When the time difference is between TimeErrors, we can simply compare by the indicators. However, it might not be fair to the earlier one with a big TimeError and low indicator. If we try to be fairer, such that letting the earlier win if the summation of the two indicators is odd, i.e., to select one randomly, it might violate the transitive law. Therefore, if we cannot make the TimeErrors the same, we need to accept some unfairness using the PowerTimestamp. The other problem might be the payload size of the needed PoW. A converted indicator such as a certificate can be verified easily first, then the details later when the PoW is ready on the receiving side. It is a traditional system tradeoff of security, performance and cost and it is feasible in real-time. Note that real-time is defined by meeting the deterministic timing constraints.

With PowerTimestamp, events can be ordered by their issuing time or sending time in the granularity of one-tenth to 1 s and the indicator with very fine granularity if users agree. We believe most users in IoT systems can be satisfied. Most side effects of the network delay can be released. As long as the blocks can arrive in time, the PowerTimestamp compares on the issuing time. Because Bitcoin protocol needs to check on some security issues at the half of the block interval, a 2-s block interval is set in OurChain to keep all timing processing in integers. Blocks can arrive in 1 s with a dedicated network connection or in local area network. Otherwise, if the users or applications need finer timing accuracy, most software can still work without modification by increasing the hardware clock rate. The PowerTimestamp is platform independent. Linux can support up to nanosecond time granularity. It is not practical for the PowerTimestamp because the network delay and the TimeError might not be smaller than nanoseconds. Therefore, the batch processing of transactions in few-second rounds is appropriate for blockchains such as OurChain using PowerTimestamp.

The last problem is faking. We can trust the indicator because it can be derived by GPoW similar to the mining process but the timestamp set in PowerTimestamp or in PoW of the PowerTimestamp can be faked. Time correction can be adopted as the NTP protocol by neighboring nodes, including some trustful NTP servers, but there might be too much overhead to verify every timestamp, and the neighboring nodes might collude altogether. Note that eclipse attack can be detected and mitigated [35] and temporary network partitioning can be tolerated. One possible ultimate solution is the hardware generated and sealed PowerTimestamp. That is, the PowerTimestamp can be real-named with the verified hardware and the timestamp can be verified later on demand. An independent and “real-named” time should not incur privacy problems. It can be implemented in the CPU or network interface card. Therefore, the PowerTimestamp can be trustful.

### 2.5. Finality

The most important reason why blockchain can provide trust is decentralization, where the artificial randomness from the hash-based PoW contributes the most. However, decentralization does not mean no centralization at all, because centralization is necessary sometimes for reasons such as politics, security, privacy, or even a centralized clock for synchronization. Actually, decentralization just tries to be as far away from centralization as possible while avoiding evil (attacks). Full decentralization is not practical and is impossible without constraints. With the PowerTimestamp, the distributed synchronization can be achieved without a centralized clock. However, for the randomness and decentralization, it is not trivial to reach the finality in blockchain because there is still a requirement from the consensus to be reached. For example, nodes with too much computing power need to be discouraged. Especially, in IoT systems, more flexibility is required. Even if some kind of consensus can be reached at a certain point of time to determine system states, the system states might be changed soon again. To be trustful, some kind of finality also needs to be reached.

Finality stands for the quality being final and impossible to be changed again. We focus on the finality of system states. Absolute, stochastic, probabilistic, and deterministic finalities have been discussed with slightly different meanings [8,19,25]. If resources are limited, system states are bounded and everything is deterministic eventually. We assume resources can be unlimited in the limited time period on consideration. Since finalities are time-related concepts, each finality can be reached and will not be changed anymore with a probability at some time after it is determined to finalize some system states in the life time of the system. The state can be final even if it is oscillating deterministically “forever”. The timestamp at this moment is a function, with a return value of bounded error of some system parameters. Actually, the function is computable as a Turing machine. In this paper, random is a synonym of stochastic and an opposite of deterministic; regardless, stochastic is on process and random is on value or result. The occurrence of random events is almost but not exactly evenly distributed. Note that a random distribution is a set of random numbers that follow a certain probability density function. It is deterministic as pseudo random number generators but not random. Those random-related terms are not mathematically defined. We simply define that it is deterministic if it can be described with a bounded error; otherwise, it is random or stochastic, including that we do not know whether it can be bounded. Probabilistic means it is under some known statistical distribution. Stochastic events happen by chance, not probabilistically. Probabilistic might not be bounded and deterministic might not be under statistical distribution. The error includes hidden stochastic events excluding unpredictable rare and big ones, i.e., out of bound or inestimable, such as a financial crisis or nuclear war. We assume deterministic events only produce deterministic effects. Some stochastic events might not have obvious stochastic effects but they will eventually be excluded from stochastic events or be classified as deterministic ones. Without loss of generality, we propose a simple definition for all the finalities, as follows.

Suppose each system state of the following finality can be reached and will not be changed anymore with a probability at some time after it is determined. The timestamp at this final moment is a function of some system parameters with a bounded error.

1. Absolute Finality: The probability is 100% and the parameters are all constant.
2. Deterministic Finality: The probability is 100% and the parameters are all deterministic.
3. Stochastic Finality: The probability or at least one of the parameters are stochastic, the value of parameter being derived random from certain algorithm or being random resulting from certain system design.
4. Probabilistic Finality: The probability is not 100% or at least one of the parameters are probabilistic. None of them are stochastic.

Since Bitcoin blocks are randomly mined and confirmed by any permissionless miners, it is with stochastic finality.

### 2.6. OurChain

OurChain [25] was based on Bitcoin with consensus PoPT based on EPoW and smart contract written in subset of C language. With the estimated computing power of remote nodes by EPoW, deterministic finality and global event ordering could be reached by PowerTimestamp. The block interval could be 2 s and the finality could be reached in 2-block epoch. The TPS could be up to 3400 under the 100 Mb/s network bandwidth. Because EPoW was still with large variance and energy consumption, PowerTimestamp and PoPT were not practically implemented. Extended from EPoW, aggressive GPoW constructs the trust model of blockchain with closed-form formula to estimate computing power to any given level of coefficient of variation. However, with only aggressive GPoW, indefinite effects occur because we do not know how many extra trials conducted. Based on conservative GPoW and aggressive GPoW, the distributed synchronization and energy consumption problems in EPoW can be solved efficiently and PoPT is feasible. Especially, with the mathematic model of conservative GPoW, statistically equivalent to aggressive GPoW, PowerTimestamp, PoPT, distributed synchronization, and global event ordering can be optimally implemented. OurChain is open-sourced and Benevolence licensed [36], supporting autonomy and sharing. Each OurChain can be an independent economy or joint economy with other OurChains for special reasons such as performance or culture. By synchronized with OurChain, other blockchains can also support deterministic finality and cross chain after 2 block intervals. OurChain based on GPoW is still in developing and will be open-sourced under the same license. It might take a long time for us to change OurChain from Bitcoin based asynchronous event-driven to PoPT synchronous time-driven for various applications. As Benevolence licensed, OurChain welcomes cooperation with the same philosophy to speed up the realization of blockchains solving disorders.

## 3. General Proof-of-Work (GPoW)

Bitcoin uses PoW with one nonce to randomly choose from valid blocks to provide trust from permissionless miners. Bitcoin nodes mine a block once a valid nonce is found. Since the nonces collected have to be valid, we might avoid the term valid before nonces later. The idea of EPoW and GPoW came from if we collected more than one nonce. EPoW extends and can estimate the computing power of miners remotely with two nonces. With the estimated computing power, EPoW can design system parameters, such as reward, accordingly to provide flexibility. Although EPoW provides a closed-form formula to estimate the computing power of remote nodes and support deterministic finality, the computation is still too complex for further analysis and can not be controlled flexibly for further needs such as the degree of confidence or trust. GPoW extends EPoW generalizing PoW with a mathematic model for mining, constructing a simple closed-form formula for further trust analysis flexibly in PoW-like blockchains. Using GPoW, at least *m* nonces need to be collected but only *m* ones, corresponding to the lowest *m* hash values, are broadcast to estimate the computing power once in each round. The variance of estimation at each round is expected to be minimized controllably. We believe this is one of the main sources of trust in the blockchain. Users can collect more than m nonces to find better *m* ones to broadcast according to different reward policies. The mining, verification and confirmation are all conducted independently to other nodes. If exactly *m* nonces are collected, it is called conservative GPoW; otherwise, it is called aggressive GPoW for more than *m* nonces collected.

**Figure 2:**
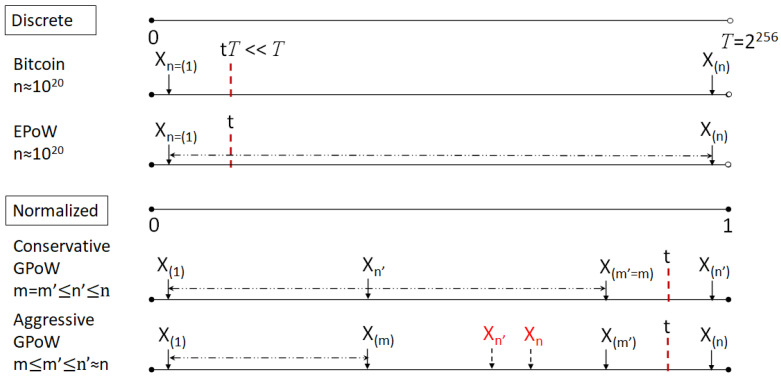
Mining by proof-of-work.

As shown in Figure 2, for mining a block, th hash values are integers independently uniformly distributed in $\left[0,T=2^{256}\right)$, ={x∈R|0≤x<T}, assuming that the cryptographic hash function is random enough. Note that the value $2^{256}$ cannot be the output of 256-bit hash functions and we follow the ISO 31-11 standard [37] to express intervals. *T* is included and normalized to a standard continuous uniform distribution in [0, 1] for easier analysis later using the beta distribution. The difference of the extra hash value can be ignored. *T* is the excluding upper bound of hash values, *t* is the normalized target value, *n* is the total number of trials, *m* is the number of required valid nonces, $n^{\prime }$ is the total number of trials when stopped earlier, and $m^{\prime }$ is the number of total valid nonces, $m\leq m^{\prime }\leq n^{\prime }\leq n$. Following the notions in Order Statistics, $X_{k}$ is the hash value of the kth trial, $X_{\left(k\right)}$ is the kth large hash value. Therefore, $X_{n=(1)}$ is the last trial, with the smallest hash value. For conservative GPoW, $m^{\prime }$ is equal to *m*. The $n^{\prime }$th trial finds the mth nonce and stops. $n^{\prime }$ might still go to *n*. For aggressive GPoW, $n^{\prime }$ goes to *n* if it is still in time. The *n* for both GPoWs can be set much smaller than that of Bitcoin or EPoW around $10^{20}$ to save energy consumption. The dashed lines stand for the range of hash values to be considered for the estimated computing power. The relative positions of *t* and the X variables are in a correct order except the red $X_{n^{\prime }}$ and $X_{n}$ with dashed arrow of aggressive GPoW. It can be anywhere between $X_{\left(1\right)}$ and $X_{\left(n\right)}$. The problem of GPoW is how to decide *t* and $n^{\prime }$, so that the system is trusted and deterministic. By adjusting *m*, the trust can be controlled to any level of granularity under physical limitation. This cannot be conducted without the mathematic proof of conservative GPoW in this paper. Similar ideas of broadcasting multiple nonces and averages of hash values are common and have been patented [38] in 2017 with only heuristics to confirm the block mined. Our work is conducted before we knew the patent. The differences are how the nonces are collected and we derive the mathematic closed-form formula for various physical meanings and applications on PowerTimestamp, PoPT, etc.

By the definition of order statistic, random variables are independent and identically distributed (IID); thus, the average of the random variables is the same IID, with the same distribution. We use the average of order statistic random variables as the indicator of estimated computing power. The lower order statistic of the indicator has the higher priority, indicating higher computing power, since the order statistic is not greater than a variable, which is exactly the target value in the context of GPoW. The lower the indicator, the more computing power it indicates. In other words, with higher computing power to try more nonces, the indicator becomes lower while the range in EPoW becomes larger. Symmetrically, the larger order statistic of the indicator can also be the higher priority. We use the lower one for fewer computations. The other feature is the time spent for the collection. It lowers the priority of the PowerTimestamp. If we make the target value big enough so that it is easy to find a nonce but still takes some time to find *m* good ones, following the requirement of PoW, much energy can be saved and the randomness of hash-based PoW could still remain. With the lowest *m* order statistics, the coefficient of variation of estimated computing power, indicating a kind of confidence of trust, can be $C=\frac{m+1}{m^{2}+m+1}$, which is explained in the following subsections. The trust indicator of this estimation can be 1−C. That is, only around m=10 trials, the trust indicator is about 90.09%. If m=1000, it can reach 99.9%. This is reasonable because common personal computer or even embedded system can reach hundreds of thousands hashes per second so that the mining overhead can be negligible.

Actually, the general PoW is a proof of PowerTimestamp (PoPT), depending on the global event ordering constructed by PowerTimestamp, using GPoW. It is a PowerTimestamp synchronized consensus. Note that there might be different kinds of trust indicators, and we just find one with simple closed-form formulas based on the PowerTimestamp. The fairest trust indicator only based on time is not available yet and might not be there unless the time is known completely.

### 3.1. Mining

The reason why Bitcoin uses hash-based PoW for mining to provide trust is that the hashing provides good randomness and fairness (with respect to computing power) to select the mined blocks to win the mining in a decentralized manner. The trustless trust (trusted by randomness so there is no need to trust any particular ones) is more trustful than just immutability, decentralization, or other blockchain features. Therefore, randomness needs to be kept in mining, especially for public blockchain. Each mining node hashes the block header with different nonces until the hash value is valid, or not greater than the target value, then the block is broadcasted for verification and confirmation.

Mining using PoW is just a way to reach consensus. There are different kinds of mining and different kinds of consensus protocols. Thus, different kinds of finality, randomness or trust can be reached to fulfill different user needs. In particular, user behavior is changing quickly with needs. With a simple and flexible metric by the formula as from GPoW, a priority mechanism can be designed as a PowerTimestamp to prioritize the needs. For example, even though the miners with higher computing power are more likely to mine a block, the total block reward in the long run might not be set exactly linear to the estimated computer power. If the block reward is set constant and the block is mined randomly as Bitcoin, in the long run, the total reward of a miner is linear to its computing power. It results in the notorious competition of computing power. According to the law of diminishing marginal returns [39], if we make the total reward to be increasing with a decreasing slope on estimated computing power, the miners with small computing power can obtain more reward than a miner with the same total computing power. It might result in Sybil attack. Thus, there are conflicts among the needs. Some AI techniques might be needed to design blockchain so that user behavior can be modeled to predict system performance, such as trust. For example, block reward is an important incentive to miner behavior and the transaction fee is to end users. Both block reward and transaction fee can be a function of the estimated computing power instead of just a constant. The function might need the parameters predicted from the distribution of user behavior at different time and location. The formulas derived in the following provide the bases from a statistical point of view to be used in real different situations.

**Figure 3:**
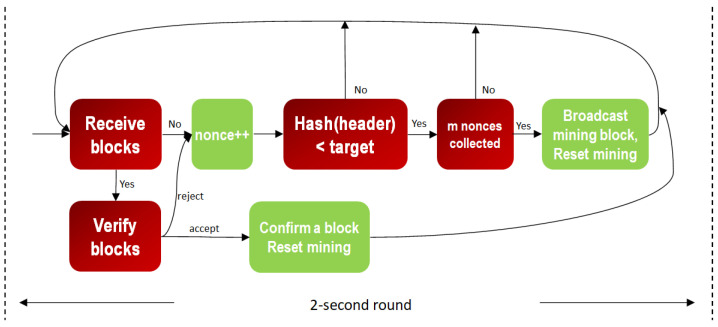
Mining flow of conservative GPoW model.

**Figure 4:**
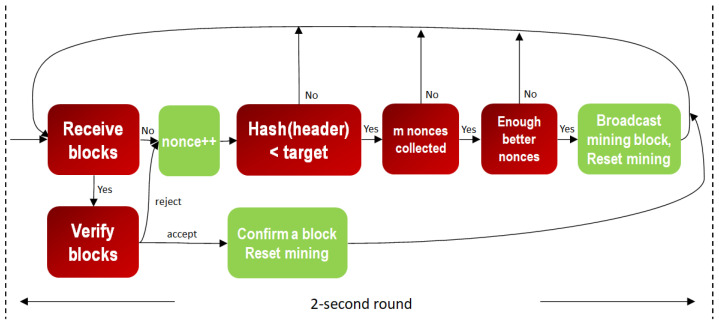
Mining flow of aggressive GPoW model.

Similar to Bitcoin mining, as shown in Figure 3 and Figure 4, miners of the two models receive, verify and confirm blocks from other miners while mining their own blocks, respectively. The difference is that each block is mined synchronously in a round of default constant period, at 2 s. When reset mining, the mined node of the confirmed block can call a smart contract to reach consensus for changing the default start time or other system parameters for the next round. The aggressive GPoW model only behaves differently from the conservative one in that it collects more than *m* nonces. Note that the nonces need to be different to be valid, as do the hash values. This is because it is required in order statistics that the sample values need to be different. It is reasonable to model GPoW because the set of 256-bit hash values is very big and the cryptographic hash function seldom collides. The problem is the number of total trials, *n*, needs to be set big enough to collect *m* nonces and small enough for the last one to be found in time for broadcasting. Although GPoW makes mining a block easy for saving energy, it might create more partitions in the blockchain network, where the PowerTimestamp is adopted as a priority to sort partitions. When reset mining for the next block, synchronization with next epoch, IoT network, cloud services, or other blockchains needs to be conducted by the PowerTimestamp. More discussion is in Section 4.

### 3.2. Conservative GPoW Mining

In a random experiment of *n* trials, the conservative GPoW collects exactly *m* nonces as a PoW in the first phase of the consensus. If m=1, it is the same as Bitcoin mining in the first phase of consensus but uses the PoW differently to estimate the computing power in the second phase. To estimate computing power, we can count statistics on consequent blocks periodically but it takes time and resources. Estimating in a block and responding accordingly in real-time, such as rejecting a block, would be more desirable. As in Bitcoin, the *m* nonces from conservative GPoW are collected in a block interval and broadcast right away when the mth nonce is found. The hash values derived from the nonces look like they are generated all at once randomly and independently, with nothing to do with estimating the computing power. Unlike aggressive GPoW, the higher computer power would push the average of the hash values lower because they are the *m* lowest ones after many extra trials before broadcasting. Since the valid hash values need to be less than the target value, with more computing power, there is still a higher probability to obtain more valid trials. Therefore, the estimation of computing power from conservative GPoW is possible, and so is Bitcoin. However, would it be inevitable with very high variance? We were not able to derive the mathematic formula for conservative GPoW but used heuristics by aggressive GPoW to estimate because, by intuition, the statistics of conservative GPoW should not be greater than the aggressive one. Besides, each cumulative distribution function (CDF) looks quite different to be equivalent. Aggressive GPoW is easier to analyze in the previous paper by the help of the Order Statistic. When trying to prove the equivalency, it is still not trivial because the same binomial coefficient nm has quite different forms, especially when n<0. It is easy to miss the equivalency without knowing they are equivalent in advance.

As shown in Figure 2, the EPoW mining is the same as Bitcoin to find a nonce with the lowest hash value, while keeping the invalid nonce with the highest hash value as the second one. EPoW tries not to change the number of trials by reusing the invalid nonces for the second one. It was mistaken in our previous paper that EPoW behaves in the same conservative GPoW mining as Bitcoin and has the same variance of the 2nd order statistic with distribution Beta (2, $n^{\prime }-1$) [25]. Actually, EPoW behaves not only as aggressive GPoW mining in the statistical point of view, with *m* = 2 and $n^{\prime }$ is the number of trials when the first valid nonce is found. It collects the invalid nonce with the highest hash value as a valid one until the real valid one is found so that the range of the two hash values might be wider, indicating the larger estimated computing power. Anyway, Bitcoin and EPoW are just special cases of GPoW. For IoT systems with limited computing power or time being critical, users might prefer to use the conservative GPoW. If the IoT devices are used in the long run, compared to frequently changing the human behavior, the conservative GPoW is more efficient and acceptable by those who care more about the average reward in the long run. We cannot apply the aggressive GPoW directly because $n^{\prime }$ is dependent on user behavior. We had to assume $n^{\prime }$ is close to *m* to be deterministic in our previous work.

For conservative GPoW with *m* nonces and *n* trials, suppose the mth nonce is found at the ith trial, and the random variable of normalized hash value is *U*. *u* is the variable of the probability of success at each trial, *t* exactly. It is the probability mass function of the negative binomial distribution when there is the mth success with i−m failures. The probability of successful *U* at the ith trial is

(1) Ui=i−1m−1um(1−u)i−m.

Because the trials stop when the mth nonce is found at the ith trial, the different ith trials look like different independent events. However, at the ith trial, if it is not successful, it can continue to the next trial. Thus, the events of conservative GPoW at the ith trial are dependent but the successful events only are still independent. Therefore, we count the independent events of success in *n* trials. By Formula (1), the probability of success in *n* trials, actually, the CDF of conservative GPoW is

(2) ∑i=mni−1m−1um(1−u)i−m

It is a new CDF. By definition, the probability density function (PDF) is the first derivative of its CDF function. Both are difficult to simplify directly for further analysis. Surprisingly, statistic formulas in aggressive GPoW can be easier simplified. We can use formulas in aggressive GPoW to analyze for conservative GPoW. Moreover, we can prove they are equivalent statistically, in the Appendix. The proof becomes simple if we believe they are equivalent no matter how they look different. Therefore, both GPoWs have the same PDF with Beta distribution and the equivalent CDF with different forms and physical meanings. The CDF of aggressive GPoW is the traditional CDF of Beta distribution, and the regularized incomplete beta function, with a physical meaning of the Order Statistic. However, the CDF of conservative GPoW can be only associated with the same regularized incomplete beta function but no other known physical meaning of statistic. Maybe we can name it the Conservative Statistic.

### 3.3. Aggressive GPoW Mining

In a random experiment of *n* trials, aggressive GPoW collects more than *m* nonces and selects *m* better ones as a PoW in the first phase of the consensus. Given any random variables $X_{1},X_{2},\ldots ,X_{n}$ with different values, the order statistics $X_{\left(1\right)},X_{\left(2\right)},\ldots ,X_{\left(n\right)}$ are also random variables, defined by sorting the values of $X_{1},X_{2},\ldots ,X_{n}$ in increasing order. For the IID random variables, the order statistics of the uniform distribution on the unit interval have marginal distributions belonging to the beta distribution family [40,41]. It is also reasonable to assume that the hash values in GPoW are IID because the cryptographic hash value is very random.

For the sample point of $X_{1},X_{2},\ldots ,X_{n}$, with CDF, $F_{X}\left(x\right)$, and PDF, $f_{X}\left(x\right)=F_{X}^{\prime }\left(x\right)$, the mth order statistics for that sample point have CDF, as follows:

(3) FX(m)(x)=∑i=mnni(FX(x))i(1−FX(x))n−i.

This is because, by definition of CDF, $F_{X}\left(x\right)=P\left(X\leq x\right)$, it is exactly the probability of success if it is successful when X≤x. Again, by definition, $F_{X_{\left(m\right)}}\left(x\right)=P\left(X_{\left(m\right)}\leq x\right)$. That is to say, $F_{X_{\left(m\right)}}\left(x\right)$ is the probability of the event that there are at least *m* variables in $X_{1},X_{2},\ldots ,X_{n}$ not greater than *x*. That is exactly the same probability of at least *m* hash values not greater than target *x* in *n* trials, the same with the aggressive GPoW. The corresponding PDF is found to be

(4) fX(m)(x)=mnmfX(x)(FX(x))m−1(1−FX(x))n−m.

Therefore, we can borrow the formula from the order statistic for aggressive GPoW mining. Denoting $U_{k}=F_{X}\left(X_{k}\right)$ to obtain the corresponding random variables $U_{1},U_{2},\ldots ,U_{n}$ from the standard uniform distribution, the order statistics also satisfy $U_{\left(i\right)}=F_{X}\left(X_{\left(i\right)}\right)$. Let $F_{U}\left(u\right)=u$ for the standard uniform distribution, so $f_{U}\left(u\right)=1$. The CDF of the mth order statistic $U_{\left(m\right)}$ is equal to

(5) ∑i=mnni(x)i(1−x)n−i.

The PDF of $U_{\left(m\right)}$ is equal to

(6) mnmum−1(1−u)n−m.

That is, the mth order statistic of the uniform distribution is a beta-distributed random variable. Actually, $U_{\left(m\right)}∼Beta\left(m,n-m+1\right)$. Formula (6) stands for the probability of doing the mth valid trial itself with exactly *m* valid trials in *n* trials. The mth trial itself will be valid 100%, so that, in the formula, it is $u^{m-1}$ instead of $u^{m}$, $u^{n-m}$ is for invalid trials, and there are mnm combinations for the mth trial. Actually, PDF is just the probability of doing the trial itself of the event at this moment to contribute to CDF no matter if it is conservative GPoW, aggressive GPoW, or something else. This is also because conservative GPoW stops after the mth valid trial. Aggressive GPoW does not need to consider more valid trials at this mth trial for $U_{\left(m\right)}$ because they are considered in other ith trials for $U_{\left(i\right)}$. Therefore, Formula (6) is exactly the same PDF in the conservative GPoW. Of course, this is explained after proved in Appendix A by formula. Now, any PDF might be derived easily without complicated mathematic proof and we can, most of the time, omit the terms conservative and aggressive before GPoW or other similar events.

Although it is not intuitive, the two models behave the same as $U_{\left(m\right)}$ from a statistical point of view. Therefore, the statistic formula in the beta distribution can be directly applied to GPoW mining. With the PDF, as in EPoW, we can estimate the computing power remotely. By statistics, GPoW mining can be mathematically described. For conservative GPoW, once *m* nonces are collected, it stops mining and broadcasts the block for confirmation. However, before it is too late to send out the mined block, miners in nature would aggressively collect more nonces for better reward. The number of extra trials to not spend too much time and lower the priority of PowerTimestamp depends on the user behavior at the same time.

**Figure 5:**
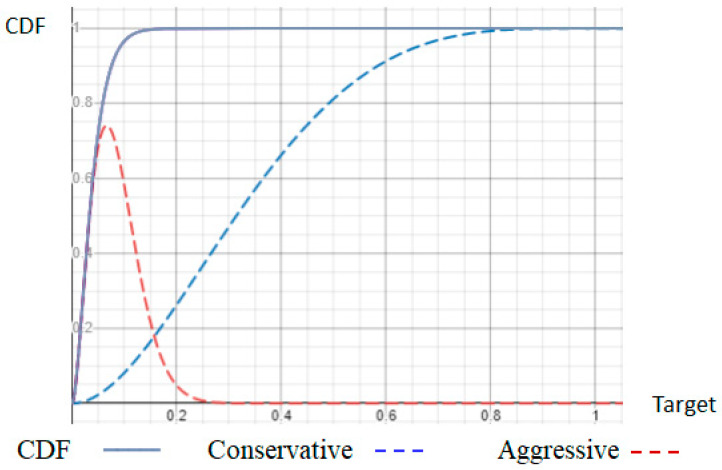
CDF is the CDF of conservative GPoW, ∑i=mni−1m−1um(1−u)i−m, and aggressive GPoW, ∑i=mnniui(1−u)n−i, overlapped, *n* = 50, *m* = 2. Conservative = ∑i=mm+3i−1m−1um(1−u)i−m and Aggressive = ∑i=mm+3ni(x)i(1−x)n−i.

We cannot tell from the same number of nonces in a block whether and how the miners are conservative or aggressive, and especially, when they have the same CDF and PDF. However, if all miners can collect more than *m* trials, that means the system is not designed collecting at least *m* nonces but at least more than *m* ones. Fortunately, the variance of various metrics becomes smaller fast when *m* is bigger. Moreover, the average of the lowest *m* order statistics among the more than *m* ones to estimate the computing power looks more advantageous for the aggressive GPoW. Actually, it is not if miners stop before the nth trial. They are equivalent only if both conduct as many *n* trials as possible in the long run. We still cannot tell whether aggressive GPoW does more than *n* trials but it is more advantageous by the mean formula of Beta distribution, $\frac{m}{n+1}$, decreasing with *n* and the lower value with the higher priority. This is one of the reasons for the intuition that aggressive work rewards more. Actually, some people aggressively work too hard in private. Moreover, if both stop before the nth trial or *n* nonces collected, it is more advantageous for aggressive GPoW when the target value is small, as shown in Figure 5, overlapped whole CDFs and the summation of CDF components for *i* from *m* to m+3, with the conservative GPoW in blue and aggressive GPoW in red. Therefore, we focus on IoT, where a faster response and less energy consumption is a common priority by setting a big target value using conservative GPoW to reach optimality if *m* nonces can be collected in time.

If we would like to make the two models behave closely, when the average estimated computing power in practice is too high than expected with planned *m* nonces, we might decrease the target value or increase *m*, periodically under consensus by a smart contract. However, it would be better fixing *m* at the design stage to avoid system overhead, such as the block header of variable sizes. The system can be designed to favor conservative or aggressive user behavior, or tuned by centralized oracles for efficiency or politics as long as the priority scheme is not changing. However, it might not be the case in the general Internet, where malicious manipulation of changing priority scheme might be easier. With a mathematic formula, the system can be quickly and systematically tuned for changing user behavior but might not be working for the priority scheme. With a consistent priority scheme as in IoT systems, for blockchain designers, they can select the *m* value and adjust the target value (or difficulty) carefully and dynamically, so that users can trust the blockchain no matter how other users might configure their system parameters autonomously or even manipulate the system maliciously.

### 3.4. Closed-Form Formula of Trust

Since the CDFs of conservative GPoW and aggressive GPoW are equivalent, by the coefficient of variation of the estimated computer power, we define the closed-form formula of trust for both by the aggressive GPoW. By the formula above borrowed from Order Statistic, we derive the following formula. By Formula (6), in the *n* trials with target *t*, the PDF of the mth order statistic $U_{\left(m\right)}$ is

(7) P(U(m)|n)=mnmum−1(1−u)n−m

and, by beta distribution, the mean $E_{U_{\left(m\right)}}\left(u\right)$ = $\frac{m}{n+1}$, the variance $V_{U_{\left(m\right)}}\left(u\right)=$$\frac{m(n-m+1)}{{\left(n+1\right)}^{2}\left(n+2\right)}$. Assume each trial is independent and will be conducted until the last one with a probability of 100%. The probability of random variable N, trial *i* happens, $P_{N}\left(i\right)=1$, uniformly distributed as used in EPoW. Note that $P_{N}\left(i\right)$ can be complicated depending on user behavior. The marginal probability of $U_{\left(m\right)}$ on N is

(8) PN(U(m))=∑i=mnP(U(m)|i)PN(i)=mum−1∑i=mnim(1−u)i−m≈mu2.

As in EPoW, by Bayes’ theorem, the probability of the ith trial at the mth order statisic of $U_{\left(m\right)}$ is

(9) P(i|U(m))=P(U(m)|i)PN(i)PN(U(m))=imum+1(1−u)i−m.

As long as $P_{N}\left(i\right)$ is a constant, $P(i|U_{\left(m\right)})$ is the same. Note that even $P_{N}\left(i\right)$ is set for simple user behavior, but the simplification for the series of summation with binomial coefficient im is not trivial. Refer to Appendix A in our previous paper [25], a special accumulation technique, for detail. Thus, the mean number of trials, linear to the estimated computing power,

(10) $$E_{U_{\left(m\right)}}\left(N\right)=\frac{m+1}{u}-1,$$

and the variance $V_{U_{\left(m\right)}}\left(N\right)=\frac{\left(m+1)(1-u\right)}{u^{2}}$. The formulas are simplified with errors removed, assuming *n* goes to infinite. Since *n*, actually $n^{\prime }$, the actual number of trials in the following, is not infinite, when estimating the computing power with $E_{U_{\left(m\right)}}\left(N\right)$ in practice, the errors might be included and the big number library is necessary. The coefficient of variation (CV) [42] $=\frac{standarddeviation}{mean}=\frac{\sqrt{variance}}{mean}$ is used as an indicator of trust because the standard deviation of data must always be understood in the context of the mean of the data. In contrast, the actual value of the CV is independent of the unit in which the measurement has been taken, so it is a dimensionless number. For comparison between data sets with different units or widely different means, one should use the coefficient of variation instead of the standard deviation. Especially, when the data are unique hash values because CV is invariant to the number of replicates while the certainty of the mean improves with the increasing number of replicates.

Therefore, the CV of the mth order statistic $CV\left(u\right)=\sqrt{\frac{n-m+1}{m(n+2)}}$, and the corresponding CV of the estimated number of trials $CV\left(N\right)=\frac{\sqrt{\left(m+1)(1-u\right)}}{m+1-u}$. The CVs depend on n, m, and t. We would like to analyze these variables so that the CVs are minimized. By definition, $E_{U_{\left(m\right)}}\left(u\right)=\frac{m}{n+1}\leq t\leq 1$. Since we do not know when the miners stop trying and both CVs increase with n and decrease with t and m, we can assign $u=t=\frac{m}{m+1}$ and n=m to bound both CVs. Thus,

(11) $$CV\left(u\right)=\sqrt{\frac{n-m+1}{m(n+2)}}\geq \sqrt{\frac{1}{m(m+2)}}$$

and

(12) $$CV\left(N\right)=\frac{\sqrt{\left(m+1)(1-t\right)}}{m+1-t}\geq \frac{m+1}{m^{2}+m+1}.$$

**Figure 6:**
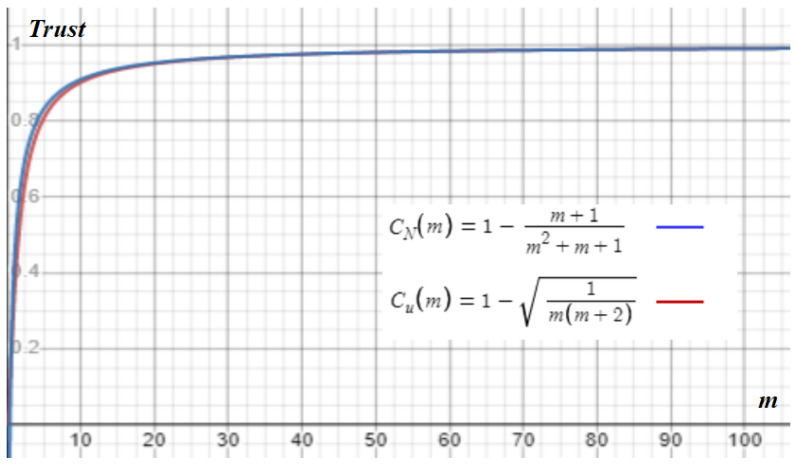
Formula of trust.

If *n* is close to *m* and *t* is close to 1, both CVs are close in value, to around $m^{-1}$, as shown in Figure 6, where $C_{u}\left(m\right)$ and $C_{N}\left(m\right)$ stand for trust indicators 1−CV(u) and 1−CV(N), respectively. However, if *n* is much larger than *m*, $C_{u}\left(m\right)$ drops a lot. Therefore, m would be better decided by the smallest integer of m when $C_{N}\left(m\right)\geq \frac{m+1}{m^{2}+m+1}$ to certain percentage such as 0.1%. Initialize target value $t=\frac{m}{m+1}$. Then, let the system adjust t periodically so that the mined blocks in a round are enough to form the highest partition easily. More accurate analysis can be conducted with actual numbers or assumptions. For example, assume miners only mine in 2 TimeErrors. However, since trust indicator $C_{N}\left(m\right)$ is in [33.3%, 57.1%, 69.2%, 80.6%, 90.0%, 99.9%] when m is in [1, 2, 3, 5, 10, 1000], respectively, it is enough for most scenarios. The transaction fee and block reward can be low in IoT systems but, for flow control, it should not be zero and might also be dynamically adjusted. This is another reason for flexible system design with the closed-form formula.

Although the IoT system might have nodes with limited computing power, some inexpensive embedded systems, such as Raspberry Pi, might still have GPU, which is much more than the GPoW needs. Actually, the IoT systems consist of machines with all kinds of computing power. The computing power of CPU and GPU mining ranges from hundreds to tens of millions of hashes per second (H/s). As of 30 September 22:00 GMT 2022, the Bitcoin network hash rate is $2.64\times 10^{20}$ H/s [43], running on about 13,912 nodes [44]. Using GPoW and aggressively mining before deadline, 2 s, suppose we use TimeError at one-tenth second, at most $n^{\prime }=10^{5}$ hashes per 2-s block for IoT nodes. With the same 13,912 nodes, we can save $\frac{2.64\times 10^{20}}{13912\times 10^{5}/2}\approx 3.795\times 10^{11}$ folds of energy consumption. Even each trial might consist of several hashes; it is still reasonable to say that GPoW reduces energy consumption to less than 1 billionth of Bitcoin, even 100 billionth. Therefore, energy consumption is negligible and software mining using the slack time in the browser or OS is possible. With limited resources, simulations for the simple configuration of GPoW are conducted with 6 nodes of CPU AMD Ryzen 9 3900X, 64GB RAM, 220K H/s, with a 2-s block interval for 200 rounds for m=2. The conservative GPoW can reach 3000 TPS in our lab networking with a bandwidth 100 Mb/s. We found it is working and the performance bottleneck is the network bandwidth. However, the 2-s block interval still has up to 0.85% errors per round after 50 rounds to be improved for the time-driven synchronization. The comparisons with other main consensus protocols [8] are listed in Table 1.

With PoPT, most attacks can be handled but nodes still might be lost in rare cases, and there might be other unknown attacks. Therefore, the fault tolerance is under 100%. The power consumption is negligible because it is less than 1 billionth of Bitcoin with the same number of nodes. The scalability is good because we can support sharding by distributed synchronization. After the parallelization of blockchain is realized, we can say it is better than other consensus protocols. Then, TPS will be limited only under physical constraints, such bandwidth or dependencies. However, Ethereum moves from PoW to PoS, reduces 99% energy consumption, and increases TPS to 2000 after upgrading to 2.0. The merge in 15 September 2022 might be subject to lower security of PoS and still needs to wait for the next upgrade to reach $10^{5}$ TPS.

Because conservative GPoW needs to include the samples of success at all trials, from mth to nth, miners do not give up before the success of the experiment. Aggressive GPoW also needs to include the samples of success with all number of nonces, from *m* to *n*; any missing would not be the mth order statistic by definition. For any target value, the same statistics of both models happen only after enough number of experiments are done. Moreover, in reality, miners stop indefinitely before the nth trial. Therefore, *n* might need to be dynamically adjusted to avoid speculation. While $n^{\prime }$ can only be measured but estimated, *n* can be initialized by the deadline to send out the block and hash rate of the node. It is not necessary for all nodes to have the same *n* value, especially when the target is close to 1 and *n* becomes not significant statistically. This might be another optimal system design factor.

**Figure 7:**
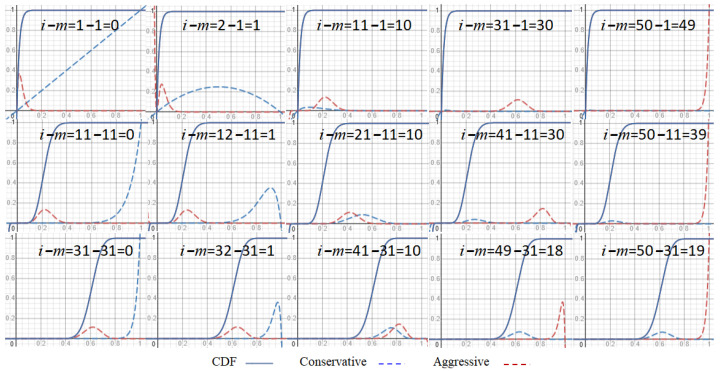
CDF is the CDF of conservative GPoW, ∑i=mni−1m−1um(1−u)i−m, and aggressive GPoW, ∑i=mnniui(1−u)n−i, overlapped, *n* = 50. Conservative = i−1m−1um(1−u)i−m and Aggressive = ni(x)i(1−x)n−i are the components of CDF, respectively. X axis is target. Y axis is CDF.

As shown in Figure 7, the two models of GPoW have the same CDF with different forms of formula overlapped with different *m* and n=50. Each formula has n−m+1 components indexed by *i* standing for the success at the ith trial in conservative GPoW or with *i* nonces collected in the aggressive GPoW. i−m stands for one set of the components, with conservative GPoW in blue and aggressive GPoW in red. Each component shapes quite differently but sums up to the same value of CDF. Conservative ones vary much more than aggressive ones. For the same *m* in a row, conservative ones have higher peaks to the left with smaller *i*, while aggressive ones have higher peaks to the right with larger *i*. For the same i−m set of components and CDFs, they shift to the right with larger *m*. Setting the target value at peaks favors the corresponding model in a short time. According to Formula (10) and u≤t, the estimated computing power varies a lot with *t*. When *t* is very small as in Bitcoin for security, and actually, m=1 with very low CDF, there is no conservative component with a high probability, and the $C_{N}\left(m=1\right)$ is at most 33.3%. That is why Bitcoin is still not trustful enough for formal applications such as CBDC. When *m* and *n* are close, we can find a target value at the intersection of the two components, one from each model. The point would be high enough so that the two models behave closely in a short time of experiments. The two models can be used in different nodes of a blockchain, even with different *m* values, especially in cross chain environment; as long as there is a mapping of estimated computing power for different *m*s, both sides agree. However, different *m* values might affect the size of nonces so do bandwidth and storage. The issue of nonce size has been discussed in our previous paper [25]. For GPoW, the nonce size has to be larger than $\left\lceil \mathrm{lg}n^{\prime }\right\rceil$ bits so that enough more than $n^{\prime }$ different trials can be generated before broadcasting a block.

## 4. Proof of PowerTimestamp (PoPT)

To reach consensus, people try many things to prove, such as work, stake, authority, bandwidth, etc. Nothing can be fairer with justice than time can prove. No chaining is needed as blockchain, time is really immutable and in no way, so far, can change the past in the real world. No one can steal time and it cannot be faked. However, we still do not know whether time is centralized or decentralized and how to use time as an instrument to define a feasible global event ordering for us to synchronize distributed events and sort any logic conflicts. These might be because time is one thing and how it is recorded as a timestamp for later use is another. If we can accept the granularity of the TimeError in PowerTimestamp, with network timestamp and estimated computing power by GPoW, a global event ordering can be defined by PowerTimestamp and the distributed synchronization for any ordered logic can be possible. This is a breakthrough when we have the equivalency of GPoWs and do not need to worry about the non-deterministic effects in aggressive GPoW using conservative GPoW. Therefore, Proof of PowerTimestamp (PoPT) is one step further than just proof of GPoW. Actually, PoPT is conceptualized earlier than GPoW but GPoW makes PoPT feasible. PoPT adopts GPoW as the first phase of consensus and designs a 2-block epoch in the second phase to avoid partitioning on the blockchain network. We explore in the following how the 2-block epoch is designed and its variants for IoT systems using PoPT. TimeError is not limited to one-tenth of a second. It can be finer depending on the network delay and computing power.

For normal Bitcoin mining, miners broadcast the mined blocks right away, without selfish mining [45], or confirm the first valid block to continue the next round of mining as soon as possible, resulting in partitioning. No matter how much computing power it saves to mine a block, any computing power needs to be utilized at the best efforts for the environment. The higher the difficulty, the less likely to mine more than 1 block concurrently for the whole network before a mined block reaches all nodes. Although the difficulty of the Bitcoin network is adjusted to mine one block in an average of 10 min, it still results in partitioning sometimes on the Bitcoin network. In other words, partitioning happens when a block is mined before another minded block arrives. Because blocks are mined randomly in the PoW blockchain, it can not prevent partitioning. The CAP theory explains this and implies that partitions can be tolerated and might be merged afterward. Deterministic finality might not be reached right away for all nodes, but it might be achieved after some deterministic time for the honest ones. Assume the honest nodes are the majority, not the majority of computing power, but the majority of nodes of the same type. We believe there exists a majority group, with affordable computing power, willing to be honest following the regulations and be able to resist collusion such as a running dummy account for the Sybil attack.

The states of a blockchain might change in any block. Some of the states might change again in the following blocks. To reach deterministic finality, it could not be conducted in a round of only one block interval. The best solution would be at least 2 block intervals for honest nodes receiving blocks normally, as the so-called 2-block epoch. PoPT uses the PowerTimestamp to synchronize all events that are not limited in OurChain, with a timestamp from NTP and estimated computing power. For any arithmetic operations of PowerTimestamps, the lower estimated computing power is kept for lower variance. The highest partition or main chain is the uniquely defined partition with the highest priority such as the most node in some grouping with the largest group ID. The group ID can be the same attribute of nodes in the group, such as the parent ID. The main chain in Bitcoin is the group of nodes with the longest chain, which is the path with the largest accumulated difficulty from genesis to the current blocks. Actually, the longest chain might not be unique. It happens when there is partitioning but it is unlikely when the current blocks become mature after 100 confirmations. However, it is still not deterministic. To tolerate partitions and merge them as soon as possible, the new mined blocks with the same grandparents are grouped in GPoW. Besides, the nodes not in the highest partition might be lost and need to rejoin the highest one in the next round. Otherwise, it needs to join as a new node. After join and rejoin, nodes are in the same main chain. Rejoin does not start from the beginning to join as long as the information it keeps is trustful. Since the computing power of all nodes can be estimated and constrained, the issuing time of the first mined block can also be bounded or referenced by the past. Each node starts next mining at the next starting time, defined one block interval after the start time of the parent block or redefined by the parent block. Thus, blocks issued too early might be malicious or speculative, and can also be rejected.

Since GPoW is synchronized with low requirement of computing power on mining, it has time to select from arriving blocks to avoid partitioning. Although technically we can not prevent Sybil attack completely, GPoW can discourage nodes with very high computing power and encourage nodes with middle class computing power forming the highest partition. Assuming the middle-class nodes can also resist more collusion and leave the nodes with low computing power intact for IoT systems if there is no special consideration such as a temporary promotion or speculation. Each node samples from the new mined blocks to estimate the highest partition. The mined block in the estimated highest partition with the highest priority by the PowerTimestamp, called the highest block, is confirmed and selected as a parent for mining next. It is simple and efficient but only probabilistic. Since the probability to mine a block is proportional to the computing power, the estimated highest partition in a node is only likely to be the highest partition in the whole network. If the estimation is not correct, the node creates a new partition in next round of mining. It will soon find itself out of the main chain in two rounds because each node needs to have the same grandparent. Therefore, it is suggested (but not necessary) that the system has more than three new mined blocks to estimate the highest partition. This can be conducted by adjusting the target value. If there happens to be no new blocks mined, a void round with no block can be constructed, forming a void parent but still with a unique ID for the next round. The target value needs to be adjusted decentralizedly in a short period of time. With the closed-form formula, it could be conducted by a smart contract in a round of the block interval, 2 s. Recall that Bitcoin is adjusted every 2016 rounds, at around two weeks. OurChain can be adjusted with a much shorter fix period or even dynamically, according to system parameters such as loading.

Any node can probe neighboring nodes to help estimating the highest partition, especially for the joining nodes or when the nodes have high partitions with close priorities. How and when to probe can be adjusted as system parameters for performance or as system features to reach different kinds of deterministic finality. Another attack is to fake on the timestamps. By adding a hash chain of timestamps for trials into PowerTimestamp and imposing time checking on neighboring nodes, timestamp-cheating mined blocks can be detected and rejected. This is another tradeoff of the performance and system feature. Assume most nodes are honest and would like to follow the consensus mechanism to maintain and merge to the main chain wherever possible. Even though there are a lot of malicious nodes conducting the Sybil attack, the system state will still be finally kept in the main chain after two block intervals. The highest block is still random and biased by the priority in the sense that the highest indicator is based on the hash-based PoW and how we estimated the computing power from the user behavior.

### 4.1. Mining Examples

It is critical to decide which nodes are in the main chain, then accept the mined blocks from these nodes for confirmation competition avoiding partitioning. However, it does not need to be with the same grandparent exactly for the highest partition. Suppose we use *s*-bit nonces, which means the ratio of the estimated computing power of the largest over the smallest can be $2^{s}$. Reward function could be exponentially decreasing and the target value could be close to 1 to encourage nodes with limited computing power. The semi-anonymous ID can be used to charge the account fee for each miner to avoid the Sybil attack. The ID is an anonymous ID linked with a real name. The real name will not be exposed unless the court orders. We would like to efficiently keep as many trustful nodes in the main chain and avoid attacks from malicious nodes. Since IoT systems are complex with different needs, the 2-block epoch might not be the only solution.

**Figure 8:**
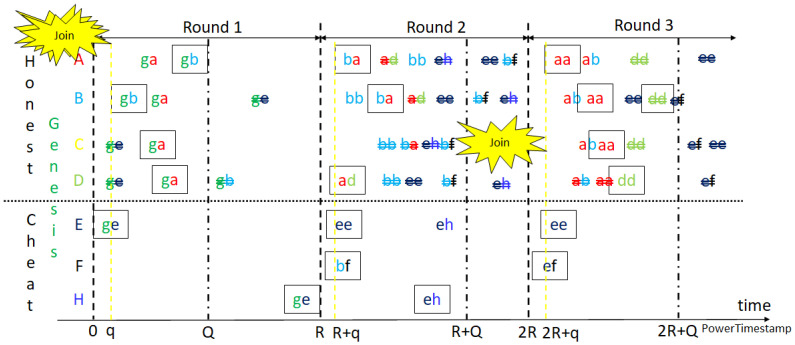
GPoW mining with partitions of the same parent.

As shown in Figure 8, suppose there are A, B, C, D, E, F, H nodes (or kinds of nodes) using PoPT in a blockchain. A, B, C, D nodes are honest. A is a remote node and B is a common node; C, with limited computing power, cannot mine a block and can just receive blocks from others. The network around the D node is not stable, where some blocks might not arrive. E, F, H nodes are malicious and they might cheat and can confirm any valid, but faked, blocks. The blocks mined or received at each node are listed after the node ID in the order of receiving or self-mined PowerTimestamps in a row, except for the cheating nodes, which only listed their own mined blocks for simplicity. For convenience, the creating time and the issuing time of a block are the same. Each block is represented by two characters. The first character indicates the parent node mined in the previous round and the second one indicates the current node in which the block is mined, in the same color of the node ID. Except in the first round, ‘g’ stands for the genesis block. The block in a rectangle is the confirmed one selected among the valid blocks in the main chain with the highest priority of the issued PowerTimestamp. The invalid blocks are crossed out. The vertical lines are the moments on each node at the time line of the PowerTimestamp to the right. Each node starts mining by joining the blockchain network at time 0 and repeats every R time units if no extra time, such as for synchronization, is needed. Blocks need to arrive between time q and Q unless the block is mined by itself. These numbers can be adjusted by the smart contract from the node of a confirmed block before the certain deadline, such as one TimeError before the new block starts, to synchronize and avoid system problems. Round X*i* stands for the Round *i* of node X.

The malicious nodes might mine a block with more than one block intervals as the bf in Round F2, faking b as the parent and not following the mining rules exactly as the honest nodes. Because the bf behaves as a new node joining after parent b, it looks fine so far. Since all blocks transmitted are with valid content, the block mined by the malicious nodes can also be confirmed. As long as the block content is valid, the blocks mined by malicious nodes should not be too harmful even after they are confirmed. Only some transactions are not included maliciously. Assuming middle-class nodes are in the majority and honest even though they might not have a higher total computing power altogether, some misbehavior by malicious nodes, such as excluding some transactions on purpose, can be resumed soon in the next few rounds by honest nodes. Therefore, some supervising mechanisms, such as detecting whether some transactions are not included in certain rounds, are needed for blockchains to protect honest users and correct misbehavior from malicious miners.

In all rounds, blocks not in time are crossed out and rejected unless they are self-mined, such as the block gb in Round D1. Suppose the nodes with the same parent node are grouped. Note that they also have the same grandparent. The blocks with different parent nodes are also crossed out as ad in Round A2 and B2 because they might be malicious. In Round 1, block ga is mined and broadcast to B, C, and D nodes. The block gb is mined but only arrives in node A in time. Node C and D do not mine a block. In node A, the blocks ga and gb are the majority. gb is confirmed because it is issued earlier. Since there is no valid block left in Round C2, it needs to join the blockchain network again. That is to probe and find the highest block ba, then copy the blockchain information back to genesis if necessary. For node D, since it did not receive block gb in Round 1, it creates another partition, such as in the malicious nodes. As long as node D can mind a block, it will not merge back to the main chain. Since it is easy to mind a block in GPoW, we lose the node D in the main chain forever unless it does not mind a block and joins again as a new node. Note that malicious nodes also form different partitions. Therefore, partitioning with the same parent can avoid the malicious attacks effectively, but it might increase the number of partitions and lose some nodes in the main chain. However, if the network is stable, this is a simple and efficient approach to confirm in one round and avoid some malicious attacks.

**Figure 9:**
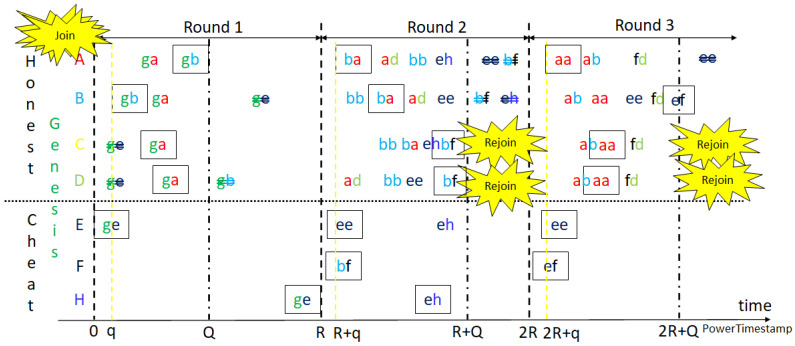
GPoW mining with all in one partition.

What if we do not group nodes to find the confirmed block but just select the one with the highest priority directly? That is, all the blocks are in the main chain. It is simple but dangerous because the malicious nodes with high computing power might dominate. As in Figure 9, all blocks including malicious ones, such as the block bf and eh in Round C3, are included in the main chain, because it is difficult to distinguish malicious ones from honest ones. Malicious blocks bf are confirmed temporarily in Round C2 and D2. The rejoin operation happens when the parent node is not consistent at Round C2, C3, D2, and D3. The main chain is resumed to the majority of honest nodes after Round 3 and the faking block bf is removed. However, if the block bf arrives in more nodes and is confirmed in Round 2, it cannot be removed. We cannot allow a block with the highest priority to dominate without any protection. On the other hand, the faked block ef in Round F3 is confirmed in Round B3. It might be more dangerous because the parent is not honest. Once it is confirmed, part of the history, including more than one block, might be replaced. However, if the nodes are trustful, normal nodes can be also confirmed in one round. Therefore, we need to group by the same grandparent to reject malicious blocks while accepting partitions caused by accident from honest nodes to some extent, leaving other partitions back to the main chain autonomously in two blocks.

**Figure 10:**
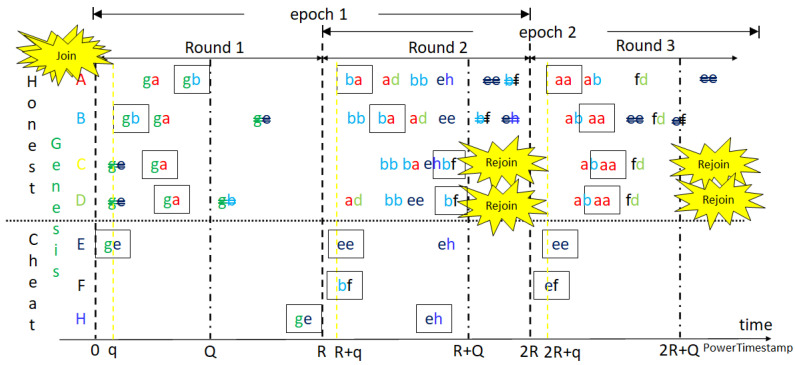
GPoW mining with 2-block epoch.

As in Figure 10, the first two rounds are the same as in Figure 9, but the blocks ee and ef in Round B3 are rejected because they are not with the same grandparent, so they are the 2-block epoch. However, the dominating problem still remains. Since we can encourage nodes within a range of the estimated computing power, we assume most nodes are in the range, forming the main chain. Because the start time is determined, beating the gun is possible if the start time can be known in advance. However, the advantage is also bounded. The malicious nodes also need to be in the range to mine a block and they need to pay more prices being evil and cannot support the majority for a long time. If we would not like to wait for a long time to solve this problem, one possible solution is narrowing the range based on some information of the blocks mined so that no one can work on purpose in advance. Then, we select the highest block from the new range for all nodes. The group of blocks in the new range can be the largest cluster in a size of close timestamps as long as it is deterministic and cannot be predicted beforehand. The size difference would better be big enough to avoid ambiguity, incurring more partitions. This approach can maintain randomness, fairness, and security to a certain extent but it cannot reach 100%. As long as the main chain is big enough in size, the other partitions, including those trustful nodes with an unstable network can join back to the main chain after two block intervals. The approaches are another system design tradeoff but only the 2-block epoch can be conducted with a deterministic finality. In other words, after two blocks, the system states of the majority nodes in the PoPT blockchain is the same and final.

### 4.2. Synchronization Scenario

In Bitcoin protocol [46], blocks are rejected if timestamp is later than two hours in the future or earlier than the median time of the last 11 blocks. Therefore, Bitcoin blocks are mined asynchronously but actually accepted in a synchronized manner. Transactions can be submitted in advance or late for about two hours for flexibility but can still reach only the stochastic finality. Solving problems of financial obligations or liquidity saving mechanisms (LSM) [47] relates to sending the transactions of payment first without enough currency in the account. We call it a liquidity-saving transaction (LSTX). There might not be enough assets or security ready to convert to the currency for payment later, either. Users who owe obligations in the circle might be settled by real-time gross settlement (RTGS) [48] without assets or security conversion but there will be a new set of transactions of the existing currency with less liquidity. We call it the LSTX set. It takes time to generate the LSTX set but can be verified quickly. The LSTX and RTGS need to be run with privacy protected. It is not trivial to support the LSM in the public blockchain for more trust. Although transactions can be conducted to be encrypted with zero knowledge proof [49] supporting privacy, it is still slow and not Turing complete [50].

**Figure 11:**
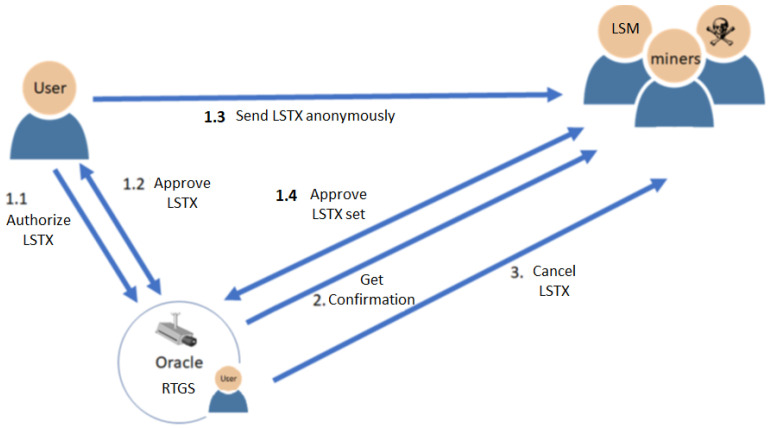
Liquidity saving transactions in blockchain.

We suggest one to run RTGS in a trusted oracle privately with public supervision and send the LSTX set later after settlement to blockchain, as shown in Figure 11. At first, LSTX is sent to the trusted oracle to obtain approval to conduct the RTGS with other LSTXs and authorize the oracle to generate the LSTX set to blockchain later, modifying the original transactions related to it. The LSTX is sent to miners anonymously even without an amount, to lock the related coin addresses from spending but also receiving. Some miners might not deal with the LSTX and some do it for VIPs with special priority, e.g., a higher transaction fee (maliciously). The miners might need to negotiate with the oracle many times to change the priority of the settlement process and adjust policies for RTGS, since the oracle only has information for the LSTX. The oracle acts as a user of the blockchain so that the negotiation can only be conducted by one-way requests. After the LSTX set is approved by both sides, the oracle obtains confirmation from miners and might need to cancel some unsettled transactions. Usually, the transactions have deadlines to be settled or canceled with the processing fee or fine involved. Therefore, without a distributed synchronization using the PowerTimestamp, it is difficult to fulfill these real-time requirements.

## 5. Conclusions

By deriving the closed-form formulas of PDF and CDF of conservative GPoW, we prove that they are equivalent, respectively, with aggressive GPoW, so that the same statistics in the long run and different behavior in a short time can be predicted and planned mathematically. Using GPoW, the PowerTimestamp is feasible. By the PowerTimestamp, a global event ordering can be achieved, and distributed synchronization is possible to reach optimality, which is a breakthrough. By designing the Proof of PowerTimestamp, the consensus PoPT can be used for the public blockchain to reach deterministic finality flexibly in real-time with less than one-billionth energy consumption of Bitcoin. Process synchronization in or cross blockchains can be also conducted in real-time under the limitation of TimeError, the maximum time error using NTP, which is one-tenth second for now. OurChain is prototyped using PoPT with GPoW of the 2-s block interval and 2-block epoch, and simulated reaching 3000 TPS. If the target value is close to 1, in the IoT system with an earlier time as a higher priority, the conservative GPoW can reach an optimality of the system requirements. We discuss examples and synchronization scenario on how to use GPoW, PowerTimestamp, and PoPT blockchain for the requirement of various IoT systems. By distributed synchronization, blockchains are platforms to support trust for IoT systems on current processing with necessary data. Most data can be kept in IoT systems and cloud services for scalability and future verification in real-time. With GPoW-supported PowerTimestamp and PoPT blockchains, trusted IoT is coming. General trusted Internet is expected if we can develop some standards to avoid the manipulation of the priority scheme after trusted IoT is mature and ubiquitous.

## Figures and Tables

**Table 1:** Comparison with main consensus protocols.

Consensus	PoPT	PoW	PoS	DPoS	PBFT	Ripple
Property						
Finality	Deterministic	Stochastic	Stochastic	Stochastic	Absolute	Absolute
Fault tolerance	<100%	50%	50%	50%	33%	20%
Power consumption	Negligible	Large	Less	Less	Negligible	Negligible
Scalability	Good	Good	Good	Good	Bad	Good
Application	Public	Public	Public	Public	Private	Private
Synchronization	OK (sharding)	Bad	Bad	Bad	Good	Good
Cross Chain	OK	None	None	None	None	None
Example	OurChain	Bitcoin	Ethereum	EOS	Eth(Istanbul)	Ripple
TPS	3000→scalable *	4–7	2000→$10^{5}$	34	2048	15

*: scalable by sharding (parallelization blockchain),→: in plan.

## Data Availability

Not applicable.

## Abbreviations

The following abbreviations are used in this manuscript:

AI	Artificial Intelligence
CAP	Consistency, Availability, and Partition tolerance
CBDC	Central Bank Digital Currency
CDF	Cumulative Distribution Function
CV	Coefficient of Variation
EPoW	Estimable Proof of Work
FLP	Fischer, Lynch, and Paterson
GPoW	General Proof of Work
IID	Independent and Identically Distributed
LSM	Liquidity-Saving Mechanisms
LSTX	Liquidity-Saving Transactions
NTP	Network Time Protocol
PDF	Probability Density Function
PoPT	Proof of PowerTimestamp
PoW	Proof of Work
RTGS	Real-Time Gross Settlement
TPS	Transaction Per Second

## Appendix A. Proof of Theorem

The following equations are used in the proof. *x* is a real number in [0, 1); the others are non-negative integers.

(A1)(1+x)n=∑k=0nnkxk nm=nn−m=n+m−1mnm−1=nn−mn−1m(A2)=nmn−1m−1=n−1m+n−1m−1(A3)∑k=0nr+kk=∑k=0nr+kr=r+n+1n(A4)−rk=(−1)kr+k−1k(A5)∑k=0nrk(−1)k=∑k=0n−r+k−1k=−r+nn

**Theorem** **A1.**
*The CDFs of conservative GPoW and aggressive GPoW are equivalent.*

**Proof.** By expanding the formula, the CDF of conservative GPoW is

(A6) ∑i=mni−1m−1xm(1−x)i−m=∑i=mni−1m−1xm∑k=0i−mi−mk(−x)k=∑i=mn∑k=i−mn−mm+k−1m−1xmki−m(−1)i−m(x)i−m=∑i=mn(∑k=i−mn−mm+k−1m−1ki−m(−1)i−m)xi.

The CDF of aggressive GPoW is

(A7) ∑i=mnnixi(1−x)n−i=∑i=mnnixi∑k=0n−in−ik(−x)k=∑i=mn∑k=0i−mni−kxi−kn−i+kk(−1)k(x)k=∑i=mn(∑k=0i−mni−kn−i+kk(−1)k)xi.

We prove by comparing the coefficients of polynomial in *x* from Formulas (A6) and (A7). If i=m+s, from Formula (A6),

(A8) ∑k=m+s−mn−mm+k−1m−1km+s−m(−1)m+s−m=∑k=sn−mm+k−1m−1ks(−1)s//byFormula(A4)=∑k=sn−mm+k−1m−1s−k−1s=m+s−1m−1−1s+m+sm−1−2s+∑k=s+2n−mm+k−1m−1s−k−1s=(s+1m+s−1−1−s+1)m+sm−1−2s+∑k=s+2n−mm+k−1m−1s−k−1s=m+s+1m+sm+sm−1−2s+m+s+1m−1−3s+∑k=s+3n−mm+k−1m−1s−k−1s=m+s+2m+sm+s+1m−1−3s+∑k=s+3n−mm+k−1m−1s−k−1s=nm+sn−1m−1m+s−n−1s.

From Formula (A7),

(A9) ∑k=0m+s−mnm+s−kn−m−s+kk(−1)k//byFormula(A5)=∑k=0snm+s−km+s−n−1k=nm+sm+s−n−10+∑k=1snm+s−km+s−n−1k=m+s−1m+snm+s−1m+s−n−11+∑k=2snm+s−km+s−n−1k=mm+snmm+s−n−1s=nm+sn−1m−1m+s−n−1s.//thesamewithFormula(A8)

If i=n, from Formula (A6),

(A10) ∑k=n−mn−mm+k−1m−1kn−m(−1)n−m=n−1m−1(−1)n−m=n−1n−m(−1)n−m//byFormula(A4)=−mn−m.

From Formula (A7),

(A11) ∑k=0n−mnn−kn−n+kk(−1)k=∑k=0n−mnk(−1)k//byFormula(A5)=−mn−m.//thesamewithFormula(A10)

Therefore, Formula (A6) and Formula (A7) are equivalent, so are the CDFs. □

Of course, the PDFs of the two models are also equivalent following the same CDFs. It can also be proved by the formula using the same techniques above. The PDF of aggressive GPoW is

(A12) ddx∑i=mnnixi(1−x)n−i=∑i=mnni(ixi−11−xn−i−(n−i)xi1−xn−i−1)=nmmxm−11−xn−m+∑i=mn−1(ni+1i+1−nin−i)xi1−xn−i−1−n−nnnxn1−xn−n−1

(A13) =mnmxm−1∑k=0n−mn−mk(−1)kxk+∑i=mn−1(nin−i−nin−i)xi1−xn−i−1=∑i=m−1n−1mnmxm−1n−mi−m+1(−1)i−m+1xi−m+1=∑i=m−1n−1mnmn−mi−m+1(−1)i−m+1xi.

Before we derive the PDF of the conservative GPoW, we need to simplify

(A14) ∑k=in−2(k+1m−1k−m+1k−i+k+1mk−mk−i)=i+1m−1i−m+10+i+1mi−m0+∑k=i+1n−2(k+1m−1k−m+1k−i+k+1mk−mk−i)=i+1mi−m+10+i+1m−1i−m+10+∑k=i+1n−2(k+1mk−mk−i+k+1m−1k−m+1k−i)=i+2mi−m+10+i+2mi−m+11+i+2m−1i−m+21+∑k=i+2n−2(k+1mk−mk−i+k+1m−1k−m+1k−i)=i+3mi−m+21+i+3mi−m+22+i+3m−1i−m+32+∑k=i+3n−2(k+1mk−mk−i+k+1m−1k−m+1k−i)=nmn−1−mn−2−i=nmn−m−1i−m+1

The PDF of the conservative GPoW is

ddx∑i=mni−1m−1xm1−xi−m=∑i=mni−1m−1(mxm−11−xi−m−(i−m)xm1−xi−m−1)=∑i=mni−1m−1xm−11−xi−m−1(m−mx−ix+mx)=∑i=mni−1m−1xm−1(m−ix)1−xi−m−1=mxm−1+∑i=m+1ni−1m−1xm−1(m−ix)∑k=0i−m−1i−m−1k(−1)kxk=mxm−1+∑i=m−1n−2(∑k=i+1n−1km−1xm−1mk−mi−m+1−1i−m+1xi−m+1−∑i=mn−1∑k=in−1km−1xm(k+1)k−mi−m−1i−mxi−m)=mxm−1+∑k=mn−1km−1mxm−1+∑i=mn−2(∑k=i+1n−1km−1mk−mi−m+1+∑k=in−1km−1(k+1)k−mi−m)−1i−m+1xi−n−1m−1nn−1−mn−1−m−1n−1−mxn−1=mnmxm−1+m∑i=mn−2(∑k=i+1n−1km−1k−mi−m+1+∑k=in−1k+1mk−mi−m)−1i−m+1xi+mnm−1n−mxn−1=mnmxm−1+m∑i=mn−2(∑k=in−2(k+1m−1k−m+1k−i+k+1mk−mk−i)+nmn−1−mi−m)−1i−m+1xi+mnm−1n−mxn−1=mnmn−m(m−1)−m+1−1(m−1)−m+1xm−1+m∑i=mn−2(nmn−m−1i−m+1+nmn−m−1i−m)−1i−m+1xi//byFormula(A14)+mnmn−m(n−1)−m+1−1(n−1)−m+1xn−1=∑i=m−1n−1mnmn−mi−m+1(−1)i−m+1xi.//thesamewithFormula(A13)

Therefore, the PDFs are also equivalent.
